# Atypical Stercoral Colitis in Chronic Opioid Use With Concurrent Hepatic and Pulmonary Lesions

**DOI:** 10.7759/cureus.108891

**Published:** 2026-05-15

**Authors:** Dhruva Govil, Fadi Ayoub, Natalie Dakki, Shymaa Hammad

**Affiliations:** 1 Internal Medicine, Henry Ford Providence Hospital, Southfield, USA; 2 Internal Medicine, Henry Ford Health System, Southfield, USA; 3 Radiology, Henry Ford Providence Hospital, Southfield, USA

**Keywords:** fecal impaction, imaging, opioid-induced constipation, rectal pain, stereococcal colitis

## Abstract

Stercoral colitis (SC) is a life-threatening inflammatory condition caused by fecal impaction and chronic opioid-induced dysmotility. While classically presenting with abdominal pain, atypical presentation can lead to diagnostic delays and increased morbidity.

A 72-year-old woman with chronic intranasal fentanyl use presented with localized rectal pain and overflow diarrhea. Initial imaging revealed SC but also identified cavitary pulmonary lesions and a hepatic mass. This constellation of findings initially raised high suspicion for disseminated malignancy. However, biopsy demonstrated inflammatory infiltrates consistent with hepatic abscess formation, raising concern for possible bacterial translocation from compromised colonic mucosa, although a definitive causal relationship could not be established. The patient was successfully managed with aggressive manual disimpaction and broad-spectrum antibiotics.

This case illustrates the diagnostic complexity of SC, where concurrent systemic inflammatory findings may mimic metastatic disease and broaden the differential diagnosis considerably. It underscores the importance of considering possible gastrointestinal sources of systemic inflammation or infection in patients with severe SC and multiorgan inflammatory findings. Recognizing overflow diarrhea is critical to avoid inappropriate anti-motility therapy, which carries a high risk of colonic perforation in the setting of SC. Early cross-sectional imaging and prioritization of reversible causes are essential when managing such complex, multisystem presentations.

## Introduction

Stercoral colitis (SC) is an uncommon but clinically significant inflammatory condition caused by fecal impaction and increased intraluminal pressure, leading to colonic ischemia and possible perforation. It most frequently occurs in patients with chronic constipation, particularly in the elderly and those with opioid use, limited mobility, or psychiatric comorbidities [1]. While SC typically presents with abdominal pain and constipation, the clinical spectrum is broad, with symptoms that can mimic various illnesses, often leading to diagnostic delay [2].

Computed tomography (CT) of the abdomen and pelvis is the preferred diagnostic modality, demonstrating fecal impaction, colonic distention, mural thickening, and pericolonic fat stranding. If untreated, SC may progress to ischemia, perforation, and sepsis, with substantial associated morbidity and mortality [3]. Management centers on prompt bowel decompression with laxatives and manual disimpaction, with antibiotics reserved for suspected or confirmed infection [4].

Although SC is primarily a gastrointestinal process, atypical presentations and concurrent systemic findings may obscure the diagnosis. In rare cases, severe mucosal compromise may theoretically permit bacterial translocation and contribute to systemic infectious complications, although direct clinical evidence remains limited [5]. We present a case of SC in a patient with chronic opioid use who developed severe rectal pain with concurrent pulmonary and hepatic lesions initially concerning for metastatic malignancy. This case highlights the diagnostic challenges posed by atypical SC presentations and demonstrates how concurrent systemic inflammatory findings may obscure the underlying gastrointestinal pathology.

## Case presentation

A 72-year-old woman with a history of pulmonary embolism on apixaban, chronic intranasal fentanyl use (~10 years), and arthritis presented with four days of severe, intermittent rectal pain. The pain was sharp, episodic, and radiated from the mid-abdomen to the rectum, lasting several minutes per episode. Her symptoms were associated with alternating constipation and diarrhea. She denied hematochezia or melena. She also reported decreased urinary output and difficulty initiating urination, attributed to poor oral intake.

One month prior to presentation, the patient had been hospitalized for *Klebsiella* bacteremia with positive blood cultures on two separate sets and was treated appropriately at that time. During the current admission, repeat blood cultures remained negative with no evidence of pulmonary or hepatic lesions.

**Figure 1:**
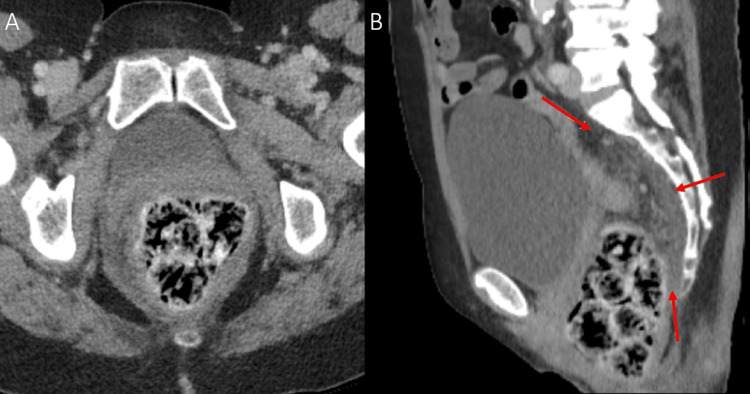
Axial (A) and sagittal (B) contrast-enhanced computed tomography (CT) of the lower pelvis. The rectum demonstrates a large rectal stool burden, mild rectal wall thickening, and peri-rectal and presacral edema and fat stranding (red arrows). On the sagittal view, the urinary bladder is distended.

On presentation, she was tachycardic and borderline hypotensive (97/68 mmHg), with otherwise stable vital signs. Laboratory evaluation revealed mild leukocytosis with neutrophilic predominance and normocytic anemia (Table 1). Initial abdominal imaging was delayed due to pain and refusal of examination. CT chest, abdomen, and pelvis ultimately demonstrated moderate colonic stool burden with rectal wall thickening, perirectal fat stranding, and surrounding fluid, consistent with SC (Figure 1). She underwent aggressive bowel decompression with bowel regimen, enemas, and manual disimpaction, with subsequent improvement in rectal pain.

**Table 1:** Laboratory values.

Name	Value	Reference range
White blood cells (WBC)	12.76 K/mcL	4-11 K/mcL
Absolute neutrophils (PMNs)	8.83 K/mcL	1.80-7.50 K/mcL
Hemoglobin (Hgb)	10.0 g/dL	12-16 g/dL

**Figure 2:**
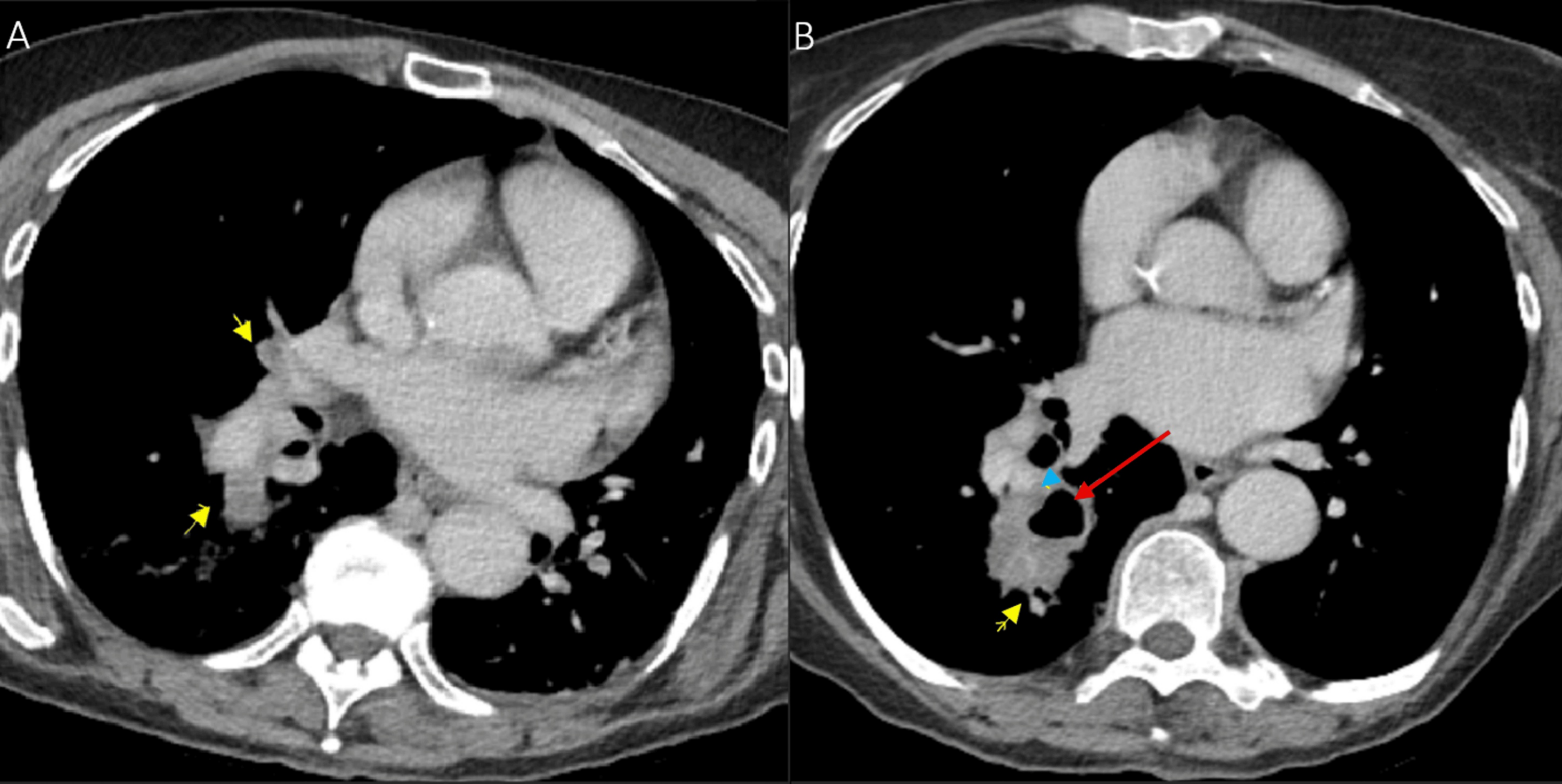
Axial contrast-enhanced computed tomography (CT), soft tissue window. (A) Multiple filling defects predominantly in the right pulmonary arteries representing pulmonary embolism. (B) Three weeks later: hypodense consolidation of the right lower lobe (yellow short arrow) with internal cavitation (red long arrow), centered upon an occluded pulmonary artery branch (blue arrowhead), concerning for septic pulmonary embolism or cavitary pulmonary infarction with superimposed infection. Interval improvement of most of the pulmonary embolism burden elsewhere, with residual linear streaks suggesting chronicity.

**Figure 3:**
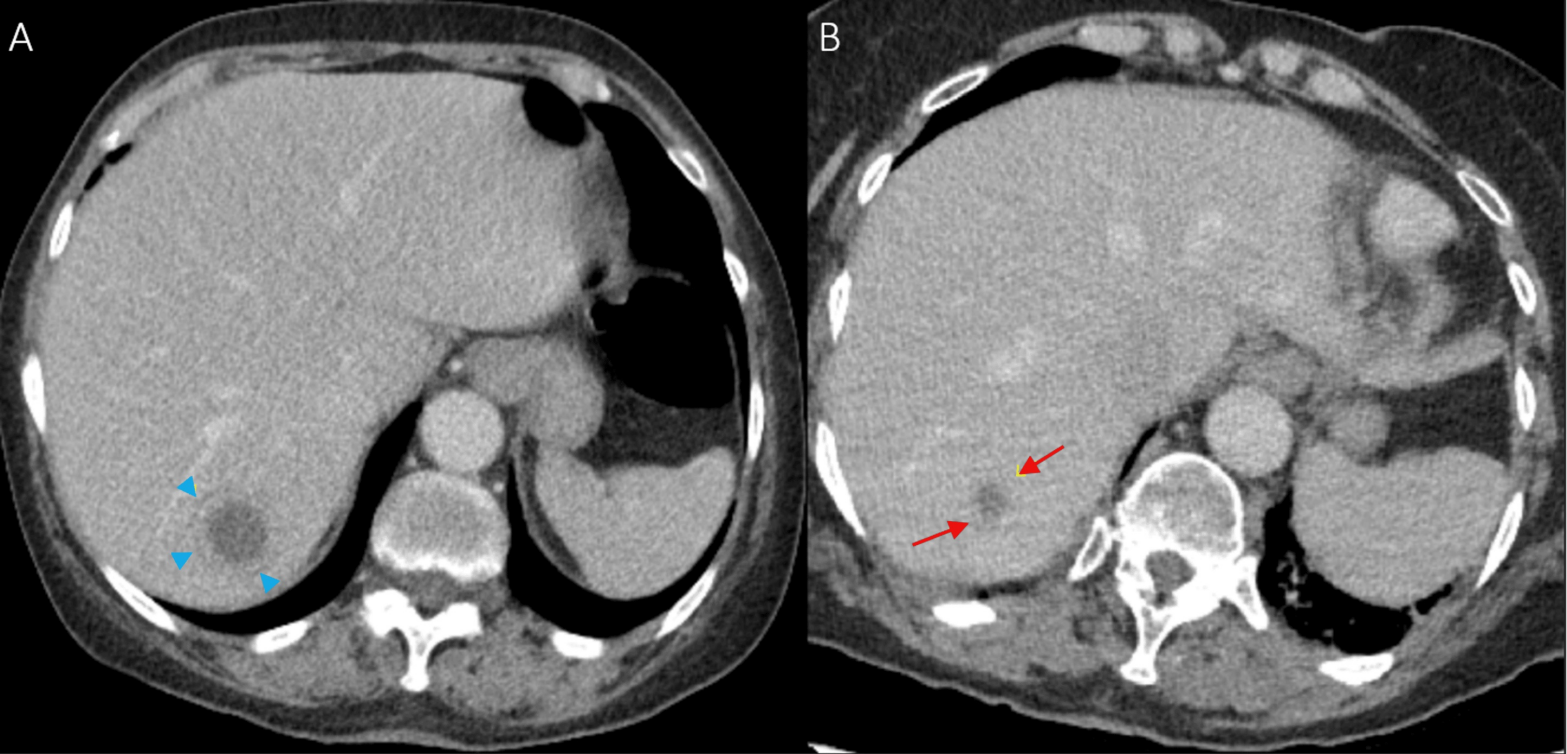
Axial contrast-enhanced computed tomography (CT) of the upper abdomen. A hypoenhancing round segment 7 hepatic lesion shows doubling in size (A) compared with the CT study obtained 3 weeks before (B, red arrows). It is surrounded by a thin hypoattenuating rim (blue arrowheads, A), after a narrow zone of enhancing parenchyma, demonstrating "double target sign." Findings are suggestive of a hepatic abscess.

Imaging also revealed additional findings, including a 5 cm cavitary right lower lobe lesion with an air-fluid level and a right upper lobe pulmonary nodule (Figure 2). A hypoattenuating right hepatic lesion measuring up to 2.7 cm was also identified (Figure 3). The constellation of these findings, cavitary lung lesions and a hepatic mass, initially prioritized a workup for disseminated malignancy versus septic emboli. Infectious diseases and oncology were consulted, and the patient was initiated on broad-spectrum antibiotics before transition to oral ciprofloxacin for a planned three-week treatment course at discharge. To further evaluate the hepatic lesion and distinguish between metastatic disease and infection, a CT-guided liver biopsy was performed, demonstrating inflammatory infiltrates consistent with abscess formation without evidence of malignancy. A bronchoscopy was also suggested but refused. Tuberculosis and fungal infectious workup were ultimately negative.

**Figure 4:**
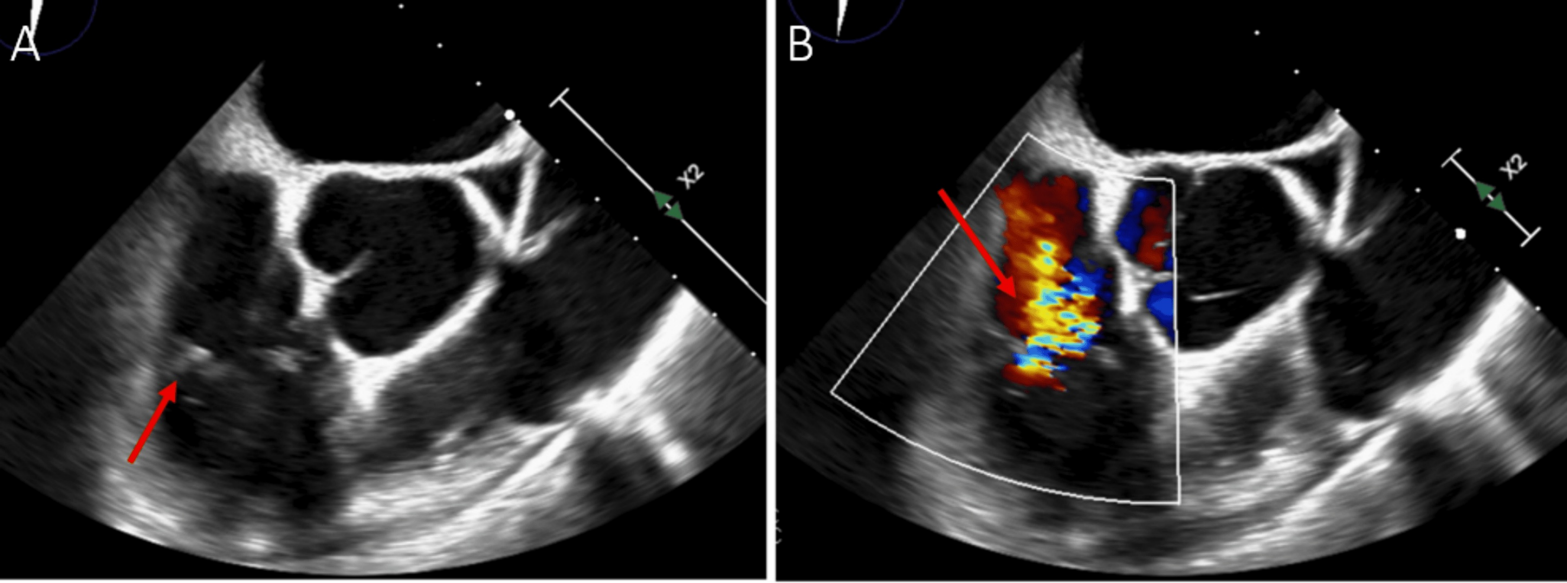
Transesophageal echocardiogram. (A) Demonstration of a thickened tricuspid valve with no evidence of endocarditis (by arrow). (B) Demonstration of moderate tricuspid regurgitation (by arrow).

Transthoracic and transesophageal echocardiography revealed a thickened tricuspid valve with moderate regurgitation but no evidence of endocarditis (Figure 4). Given clinical stability and improvement in gastrointestinal symptoms following disimpaction, the patient was discharged with close outpatient follow-up for infectious, pulmonary, and hematologic evaluation. At outpatient follow-up, the patient reported continued improvement in gastrointestinal symptoms without recurrence of severe rectal pain.

## Discussion

The clinical significance of this case lies in the diagnostic complexity created by the coexistence of SC with concurrent pulmonary and hepatic inflammatory lesions. While the association between chronic opioid use and SC is well-documented, the presentation of severe, localized rectal pain and overflow diarrhea, rather than diffuse abdominal pain, can lead clinicians toward a primary anorectal or infectious diarrhea workup, delaying the recognition of the underlying fecaloma. Notably, prior studies suggest that up to 25%-60% of patients with SC may lack classic abdominal pain [6].

SC results from prolonged fecal stasis leading to increased intraluminal pressure and mucosal ischemia with risk of perforation [7]. Chronic opioid use significantly exacerbates this process through μ-opioid receptor-mediated inhibition of motility and increased fluid absorption, resulting in opioid-induced constipation (OIC) [6]. In this patient, long-standing intranasal fentanyl use likely played a central role in the development of fecal impaction and subsequent SC.

The most challenging aspect of this case was the presence of concurrent pulmonary and hepatic lesions, which substantially broadened the differential diagnosis to include disseminated malignancy, septic emboli, fungal infection, mycobacterial infection, and infected pulmonary infarction. While the causal link between SC and distant abscesses cannot be definitively proven in every instance, the pathophysiologic mechanism of bacterial translocation via pressure-induced mucosal injury remains biologically plausible and has been described in severe colonic inflammatory states [8]. Disruption of mucosal integrity in severe colonic inflammation has been shown to allow translocation of enteric bacteria into the portal circulation, potentially contributing to hepatic infectious complications in select cases [3,8,9]. In the absence of endocarditis and with negative tuberculosis and fungal evaluation, the gastrointestinal tract remained a possible source of systemic inflammation or infection. However, a definitive causal relationship between SC and the pulmonary and hepatic lesions could not be conclusively established.

This case also emphasizes the importance of recognizing overflow diarrhea as a manifestation of fecal impaction. Misinterpretation of these symptoms may delay appropriate bowel-directed therapy and prolong morbidity. CT imaging remains the diagnostic modality of choice, with findings including fecal impaction, colonic dilation, mural thickening, and pericolonic fat stranding [7]. Early recognition is critical, as progression to ischemia or perforation carries a mortality rate of up to 35% [10].

Management of such cases requires a shift from purely conservative care to a multidisciplinary approach that addresses potential systemic seeding. In the absence of peritonitis, manual disimpaction is effective; however, pain management in the setting of opioid use disorder presents a therapeutic paradox. Escalating opioids for SC-related pain may further impair gastrointestinal motility and worsen fecal stasis, complicating clinical management. A multidisciplinary approach is therefore essential to balance analgesia with minimization of further bowel dysfunction [6].

Overall, this case underscores how SC can present with localized, misleading symptoms and coexist with systemic infectious findings that complicate diagnosis. Recognition of both atypical presentations and potential extraintestinal infectious complications is essential to prompt timely evaluation, avoid unnecessary invasive workup, and prevent progression to life-threatening outcomes.

## Conclusions

SC is an underrecognized but potentially severe complication of chronic constipation, particularly in patients with chronic opioid use. This case demonstrates how atypical features such as predominant rectal pain and overflow diarrhea may obscure the diagnosis, especially when concurrent pulmonary and hepatic lesions broaden concern for disseminated malignancy or systemic infection. Although a definitive causal relationship between SC and the extraintestinal findings could not be established, the case highlights the importance of maintaining a broad differential diagnosis and considering gastrointestinal pathology in patients with unexplained multiorgan inflammatory findings. Early cross-sectional imaging, prompt bowel decompression, and multidisciplinary evaluation remain essential to preventing progression to ischemia, perforation, and other serious complications.

## References

[1] Bae E, Tran J, Shah K (2024). Stercoral colitis in the emergency department: a review of the literature. Int J Emerg Med.

[2] Lee F, Cao J, Lin E, Kurashima M, Okeke RI, Saliba C, Miyata S (2023). The extremes of constipation: a case of stercoral perforation from fecal impaction in a teenager. Cureus.

[3] Ünal E, Onur MR, Balcı S, Görmez A, Akpınar E, Böge M (2017). Stercoral colitis: diagnostic value of CT findings. Diagn Interv Radiol.

[4] Ahmad H, Jannat H, Khan U, Ahmad N (2023). Stercoral colitis: a diagnostic challenge and therapeutic approach in an elderly patient with chronic constipation. Cureus.

[5] van Nispen C, Long B (2025). High risk and low incidence diseases: stercoral colitis. Am J Emerg Med.

[6] Farmer AD, Holt CB, Downes TJ, Ruggeri E, Del Vecchio S, De Giorgio R (2018). Pathophysiology, diagnosis, and management of opioid-induced constipation. Lancet Gastroenterol Hepatol.

[7] Maurer CA, Renzulli P, Mazzucchelli L, Egger B, Seiler CA, Büchler MW (2000). Use of accurate diagnostic criteria may increase incidence of stercoral perforation of the colon. Dis Colon Rectum.

[8] Berg RD (1995). Bacterial translocation from the gastrointestinal tract. Trends Microbiol.

[9] Ronan MV, Herzig SJ (2016). Hospitalizations related to opioid abuse/dependence and associated serious infections increased sharply, 2002-12. Health Aff (Millwood).

[10] Wu CH, Wang LJ, Wong YC (2011). Necrotic stercoral colitis: importance of computed tomography findings. World J Gastroenterol.

